# Supplementary material for: A viral fusogen hijacks the actin cytoskeleton to drive cell-cell fusion
Source: eLife. 2020 May 22;9:e51358. doi: 10.7554/eLife.51358 (PMC7244324; doi:10.7554/eLife.51358)
Supplement: Supplementary file 1. [file elife-51358-supp1.docx]

| **Key Resources Table** | | | | |
| --- | --- | --- | --- | --- |
| **Reagent type (species) or resource** | **Designation** | **Source or reference** | **Identifiers** | **Additional information** |
| cell line (*Homo-sapiens*) | HEK293T | UCSF Cell culture facility | RRID:CVCL_1926 |  |
| cell line (*M. musculus*) | SYF | ATCC | Cat# CRL-2459, RRID:CVCL_6461) |  |
| cell line (*M. musculus*) | SYF + c-src | ATCC | Cat# CRL-2498, RRID:CVCL_8976 |  |
| cell line (*M. musculus*) | N-WASP  -/- mouse embryonic fibroblast | Scott Snapper | N-WASP -/- |  |
| cell line (*M. musculus*) | N-WASP +/+ mouse embryonic fibroblast | Scott Snapper | N-WASP +/+ |  |
| strain, strain background (*Escherichia coli*) | Rosetta (DE3) | EMD Millipore/ QB3 Macrolab |  |  |
| recombinant DNA reagent | pcDNA3.1 (+) (plasmid) | Invitrogen |  |  |
| sequence-based reagent | p14 | Integrated DNA Technologies | Accession #: Q80FJ1 | Membrane fusion protein synthesized as gBlock |
| transfected construct (Reptilian orthoreovirus) | pcDNA-p14-WT-mCherry (plasmid) | This paper; Figure 1. |  | Construct to transfect and express p14 with Kozak sequence and C-terminus mCherry tag |
| transfected construct (Reptilian orthoreovirus) | pcDNA-p14-Δcyto-mCherry (plasmid) | This paper; Figure 1. |  | Construct to transfect and express p14 with ΔQ70-I125 with C-terminus mCherry tag |
| transfected construct (Reptilian orthoreovirus) | pcDNA-p14-Δecto-mCherry (plasmid) | This paper; Figure 5. |  | Construct to transfect and express p14 with ΔM1-T35 with C-terminus mCherry tag |
| transfected construct (Reptilian orthoreovirus) | pcDNA-p14-Y59F-mCherry (plasmid) | This paper, Figure 2. |  | Construct to transfect and express p14 mCherry with Y59F mutation. |
| transfected construct (Reptilian orthoreovirus) | pcDNA-p14-Y77F-mCherry (plasmid) | This paper, Figure 2. |  | Construct to transfect and express p14 mCherry with Y77F mutation. |
| transfected construct (Reptilian orthoreovirus) | pcDNA-p14-Y96F-mCherry (plasmid) | This paper, Figure 2. |  | Construct to transfect and express p14 mCherry with Y96F mutation. |
| transfected construct (Reptilian orthoreovirus) | pcDNA-p14-Y100F-mCherry (plasmid) | This paper, Figure 2. |  | Construct to transfect and express p14 mCherry with Y100F mutation. |
| transfected construct (Reptilian orthoreovirus) | pcDNA-p14-Y116F-mCherry (plasmid) | This paper, Figure 2. |  | Construct to transfect and express p14 mCherry with Y116F mutation. |
| transfected construct (Reptilian orthoreovirus) | pcDNA-p14-N118A-mCherry (plasmid) | This paper, Figure 2. |  | Construct to transfect and express p14 mCherry with N118A mutation. |
| transfected construct (Reptilian orthoreovirus) | pcDNA-p14-FVAI-mCherry  (plasmid) | This paper, Figure 2. |  | Construct to transfect and express p14 mCherry with Y116F/ N118A mutations. |
| recombinant DNA reagent | pLV-EF1a-IRES-Puro (plasmid) | Addgene, Tobias Meyer | Plasmid #: 85132 | Lentiviral transfer plasmid with IRES Puro for selectio |
| recombinant DNA reagent | pHR-IRES-Puro (plasmid) | This paper, Figure 4. |  | Lentiviral transfer plasmid with IRES Puro for selectio |
| transfected construct (*Homo-sapiens*) | pHR-N-termSH3-IRES Puro  (plasmid) | This paper, Figure 4. |  | Lentiviral construct to express Grb2 N-terminus SH3 domain and SH2 domain (1-159) |
| transfected construct (*Homo-sapiens*) | pHR-C-termSH3-IRES Puro  (plasmid) | This paper, Figure 4. |  | Lentiviral construct to express Grb2 C-terminus SH3 domain and SH2 domain (58-217) |
| transfected construct (*Homo-sapiens*) | pHR-SH2-IRES Puro  (plasmid) | This paper, Figure 4. |  | Lentiviral construct to express Grb2 SH2 domain (58-159) |
| transfected construct (*Homo-sapiens; eneterohemorrhagic E. coli*) | pHR-SH2R47-IRES Puro  (plasmid) | This paper, Figure 5. |  | Lentiviral construct to express 47 residues from EpsF(U) of enterohemorrhagic E. coli (268–314) ligated with GGGS linker downstream of Grb2 SH2 domain |
| recombinant DNA reagent | pBiFC-bJun-YN155 | Tom Kerppola |  |  |
| recombinant DNA reagent | pBiFC-bFos-YC155 | Tom Kerppola |  |  |
| transfected construct (*Homo-sapiens*) | pHR-splitYFPa (plasmid) | This paper, Figure 1. |  | Lentiviral construct to express splitYFPa |
| transfected construct (*Homo-sapiens*) | pHR-splitYFPb (plasmid) | This paper, Figure 1. |  | Lentiviral construct to express splitYFPa |
| transfected construct (*G. gallus*) | pLNCX chick src Y527F (plasmid) | Addgene, Joan Brugge | Plasmid #13660 |  |
| transfected construct (*G. gallus*) | pcDNA-CAsrc-FLAG (plasmid) | This paper, Figure 2. |  | Construct to transfect and express constitutively active c-src (Y527F) with C-terminus FLAG tag |
| transfected construct (*G. gallus*) | pcDNA-CAsrc-BFP (plasmid) | This paper, Figure 3. |  | Construct to transfect and express constitutively active c-src (Y527F) with C-terminus mTagBFP2 |
| transfected construct (*G. gallus*) | pcDNA-KDsrc-FLAG (plasmid) | This paper, Figure 2. |  | Construct to transfect and express kinase dead c-src, (Y527F/K295R)with C-terminus FLAG tag |
| transfected construct (*G. gallus*) | pcDNA-KDsrc-BFP (plasmid) | This paper, Figure 3. |  | Construct to transfect and express kinase dead c-src, (Y527F/K295R)with C-terminus mTagBFP2 |
| chemical compound, drug | TransIT-293 | Mirus Bio | Cat #: MIR-2700 |  |
| chemical compound, drug | CellMask™ Deep Red Plasma membrane Stain | Thermo Fisher Scientific | Cat#: C10046 | 1:2000 |
| chemical compound, drug | Hoechst 33342 | Life Technologies | Cat#: H3570 | 1:300 |
| chemical compound, drug | Sorafenib tosylate | Selleckchem | Cat#: S1040 |  |
| chemical compound, drug | CK-666 | Sigma Aldrich | Cat#: sml0006 |  |
| chemical compound, drug | Wiskostatin | Sigma Aldrich | Cat#: w2270 |  |
| chemical compound, drug | smifH2 | Calbiochem | Cat#: 344092 |  |
| chemical compound, drug | PhosSTOP phosphatase | Roche | Cat#: 04906845001 |  |
| chemical compound, drug | HALT protease inhibitor | Thermo Fisher Scientific Scientific | Cat: PI-87785 |  |
| commercial assay or kit | GFP-Trap Magnetic Agarose | Chromotek | Cat#: gtma20 |  |
| commercial assay or kit | Protein-G Dynabeads | Thermo Fisher Scientific Scientific | Cat: 10003D |  |
| antibody | α-FLAG (mouse, monoclonal) | Sigma Aldrich | Cat# F1804, RRID:AB_262044 | Wb (1:5000), IP (3 μg) |
| antibody | α-Grb2 (mouse, monoclonal) | BD Biosciences | Cat# 610111, RRID:AB_397517 | Wb (1:5000) |
| antibody | α-tubulin (rat, monoclonal) | Thermo Fisher Scientific | Cat# MA1-80017, RRID:AB_2210201 | Wb (1:5000) |
| antibody | α-pTyr (rabbit, polyclonal) | Cell Signaling Technology | Cat# 8954, RRID:AB_2687925 | Wb (1:2000) |
| antibody | α-GFP (mouse, monoclonal) | Molecular Probes | Cat# A-11120, RRID:AB_221568 | Wb (1:10,000) |
| antibody | α-GFP (rabbit, polyclonal) | Sigma Aldrich | Cat# G1544, RRID:AB_439690 | Wb (1:5000) |
| antibody | α-mouse HRP (donkey, polyclonal) | Jackson ImmunoResearch Labs | Cat# 715-035-151, RRID:AB_2340771 | Wb (1:5000) |
| antibody | α-mouse HRP | Upstate Biotechnology |  | Wb (1:10,000) |
| antibody | α-rabbit HRP (goat, polyclonal) | Thermo Fisher Scientific | Cat# 65-6120, RRID:AB_2533967 | Wb (1:5000) |
| antibody | α-rat AlexaFluor 647 (goat polyclonal) | Molecular Probes | Cat# A-21247, RRID:AB_141778 | Wb (1:5000) |
| antibody | α-biotin-AF647 (mouse, monoclonal) | Santa Cruz Biotechnology | Cat# sc-53179, RRID:AB_628779 | Wb (1:5000) |
| recombinant DNA reagent | pGEX4T2 | GE Healthcare Life Sciences |  |  |
| sequence-based reagent | Grb2 cDNA clone (*Homo-sapiens)* | GE Dharmacon | Clone ID: 3345524 |  |
| recombinant DNA reagent | pGEX4T2-Grb2 | This paper, Figure 3. |  | Construct to express GST-Grb2 in *E. coli* |
| peptide, recombinant protein | TEV protease | QB3 Macrolabs |  |  |
| peptide, recombinant protein | N-WASP (ΔEVH1) | D. Wong and J. Taunton |  |  |
| peptide, recombinant protein | Capping protein | S. Hansen and D. Mullins |  |  |
| peptide, recombinant protein | Arp2/3 | Cytoskeleton Inc. |  |  |
| peptide, recombinant protein | Profilin | Bieling et al., 2016. (PMID: 26771487) |  |  |
| peptide, recombinant protein | Actin | Spudich & Watt, 1971 (PMID: 4254541) |  | Rabbit skeletal muscle actin |
| peptide, recombinant protein | Cofilin | Bieling et al., 2016. (PMID: 26771487) |  |  |
| peptide, recombinant protein | Utrn-AF488 | Bieling et al., 2016. (PMID: 26771487)  Harris et al., 2019. (doi: 10.1091/mbc.E19-06-0317) |  | Utrophin actin binding domain (1-261) |
| commercial assay or kit | Streptavidin polystyrene beads | Bangs Laboratoies | Cat#: CP01N |  |
| peptide, recombinant protein | Biotin-p14 cytoplasmic tail (pYVNI) | Genscript |  | Biotin-KPPPpYVNIDN |
| peptide, recombinant protein | Biotin-p14 cytoplasmic tail (YVNI) | Genscript |  | Biotin-KPPPYVNIDN |
| commercial assay or kit | EZ-Link Sulfo-NHS-Biotin | Thermo Fisher Scientific | Cat #: A39256 |  |
| Software | Micro-manager | NIH/ Edelstein et al., 2014 (doi: 10.14440/jbm.2014.36) |  |  |
| Software | ImageJ | NIH |  |  |
