# Supplementary material for: A viral fusogen hijacks the actin cytoskeleton to drive cell-cell fusion
Source: eLife. 2020 May 22;9:e51358. doi: 10.7554/eLife.51358 (PMC7244324; doi:10.7554/eLife.51358)
Supplement: Peer review history

# Decision letter

**DOI:** 10.7554/eLife.51358.sa1

**Affiliations:** Tel Aviv UniversityIsrael; Technion-Israel Institute of TechnologyIsrael; NICHD, NIHUnited States

In the interests of transparency, eLife publishes the most substantive revision requests and the accompanying author responses.

**Acceptance summary:**

The work explores mechanisms of cell fusion mediated by the intriguing protein fusogens of the FAST family of reovirus fusion proteins. The authors develop the hypothesis that these proteins promote close contact between cells and membrane fusion by controlling local rearrangements of actin cytoskeleton, and they present several well-conducted experiments in support of this hypothesis. However, additional future experiments, as suggested by one of the reviewers, will be needed to further clarify the mode by which this viral fusogen hijacks the host's cytoskeleton to drive cell-cell fusion.

**Acceptance letter:**

We include below the comments and reservations of one of the reviewers, which the authors might find useful for their future work and presentation of their results.

Some experiments showing Wb following surface biotinylation show that there is more wildtype p14 on the surface than in the mutants (for example cytotail delletion in Fig 1S1g & h) show lower expression and reduced fusion. If they had reduced the concentration of p14 by using lower DNA concentrations in the transfection or by inducing down regulation it would have been more convincing.

For example, Y116F and tailless mutant appear to have lower amounts of protein on the surface that correlates with reduced activity. Thus, it is not convincing that "trafficking to the plasma membrane was intact" for the different mutants and under the different treatments.

While they show that CK-666 treatments resulted in a dose-dependent reduction in fusion, they did not show what is the surface expression of p14 on the plasma membrane at different dosages. Fig 4S1a shows a reduction in surface expression compared with wt. In addition, it is not clear how they normalized for loading. Using cadherin, integrin or another surface protein could help here.

Response Figure 2B shows a reduction on the percentage of p14 expressing cells.

Response Figure 1A shows lower surface expression for some mutants that had lower fusion. The authors did not have loading controls and the surface expression was not normalized. In summary, using increasing amounts of DNA could have been used to try to correlate surface expression with fusion.

Knock down or knock out experiments: The authors were unable to show that N-WASP can be exchanged by a different actin nucleator. They did not show whether WASP(-/-) affects the surface expression of p14. They could have rescued N-WASP(-/-) instead of comparing with WT MEFs. Experiments on Grb2 shRNA and KO were not conclusive.

Do actin protrusions correlate with p14 localization and fusion?

The authors did try to look for p14 and actin filaments and networks at fusion sites. However, actin protrusions were not observed and the colocalization of p14 with actin fibers and branched actin was not shown to correlate with fusion. It appears that the proteins used in this study are highly overexpressed and the resolution of the colocalization studies is very low. Thus, it is not convincing that in cells p14 colocalizes with actin cytoskeleton and actin binding proteins. The comparison of the results of this study to the Listeria model is not convincing.

**Decision letter after peer review:**

Thank you for submitting your article "A viral fusogen hijacks the actin cytoskeleton to drive cell-cell fusion" for consideration by *eLife*. Your article has been reviewed by Anna Akhmanova as the Senior Editor, a Reviewing Editor, and four reviewers. The following individual involved in review of your submission has agreed to reveal his identity: Benjamin Podbilewicz (Reviewer #1).

The reviewers have discussed the reviews with one another and the Reviewing Editor has drafted this decision to help you prepare a revised submission.

Summary:

This study by Ka Man Carmen Chan et al., focused on one of the most intriguing examples of membrane fusion, cell-cell fusion mediated by FAST proteins of non-enveloped reoviruses such as the reptilian orthoreovirus p14. The authors characterized the interactions between p14 and the actin cytoskeleton, showed that c-Src kinase can phosphorylate a specific tyrosine (Y116) on the cytoplasmic tail of p14, which can lead to interaction of this cytotail with the cellular adaptor protein Grb2. They further showed that in vitro this interaction can initiate the interaction with N-WASP nucleation factor that can drive localized assembly of the branched actin networks mediated by Arp2/3 complex.

The central message of the work, according to the current presentation of the results, is a proposal of a novel mechanism by which the polymerizing actin network is crucially involved in the process of membrane fusion. Since p14 is very short compared to the best characterized fusogens, and, therefore, could not reach the target membrane, the authors suggest that the primary role of the branched actin network assembly is to develop a force applied to the p14 ectodomain and push it into a close contact with a neighboring cell.

All the reviewers including the reviewing editor found the study to be elegant, the experiments well designed, and the article well written.

The reviewers agreed that the achievements of the work are:

a convincing demonstration of the p14-regulated assembly of branched actin, dissection of the way p14 regulates this assembly via p14/Grb2/N-WASP interactions, and demonstration of the importance of this assembly for p14-mediated fusion.

At the same time, the reviewers were not convinced that the proposed mechanism for the role of actin polymerization in p14-mediated fusion, which is presented by the authors as the major result of the work, was sufficiently supported by the presented data, and found it largely hypothetical. It is the opinion of the majority of the reviewers that substantiation of the mechanism is crucial for acceptance of the article for publication in *eLife*.

Essential revisions:

The first group of questions, which have to be addressed, is related to the uniqueness of the proposed mechanism. Specifically, can N-WASP mediated assembly of a branched actin network promote fusion by raising local concentration of p14 at the future fusion site? Can one exclude fusion dependence on branched actin dependent cell-cell adhesion (Efimova and Svitkina, 2018)? Is there any evidence (perhaps in the literature) that fusion depends on interactions between p14 ectodomain and the target membrane rather than on the p14 ectodomain interactions with the p14-expressing membrane? Can one decrease the dependence of p14-mediated fusion on the assembly of the branched actin networks by "shaving" the surface of the target cells with proteases?

The reviewers realize that some of these questions are most likely beyond the scope of this study so that they can be addressed by mentioning alternative interpretations and avoiding the definitive language such as "we demonstrate" while discussing the proposed mechanism.

At the same time, one of the alternative mechanisms has to be ruled out experimentally, namely, the mechanism in which the factor controlling fusion is the local concentration of p14, whereas the role of the polymerizing actin network is to modulate this concentration rather than to develop a pushing force. To address that point the reviewers suggest to:

1) Quantify the surface expression of p14 in wt and mutants under different experimental conditions and demonstrate that the differences in the fusion efficiency do not correlate with those in the p14 concentrations.

Addressing this point is especially important since differences in the number and density of fusogens on the surface can result in different levels of fusion. This has been shown for many fusogens including viral (e.g. Influenza's HA), exoplasmic (e.g. EFF-1) and endoplasmic (e.g. SNAREs). One of the reviewer suggested that the authors do surface biotinylation on ice for all the mutants under different pharmacological conditions (for example in Figure 1F, Figure 1—figure supplement 1B, Figure 2C, Figure 3B,C,E, Figure 4B, Figure 5A).

2) Perform knock down or knock out experiments using siRNAS or CRISPR-Cas9 in order to determine specifically whether Grb2, N-WASP, Arp2/3, Raf kinase and Ras are required for p14-mediated fusion.

3) Perform cell-cell imaging and colocalization of p14, actin and contact sites between membranes before during and after fusion can help to test the model.

The second group of questions is concerned with the physical background of the mechanism by which polymerizing actin could exert a force sufficient to press p14 ectodomains against the target membrane.

As an experimental substantiation of the mechanism the reviewers expect a test where, for example, another nucleator is used, so that the actin network (and, hence, the force) that pushes the protein is changed. In addition, p14 could be made longer, with repetitive motifs, to see whether the distance between the protein ends matters.

On the theoretical level, the way the force pushing p14 is generated is not clear. Indeed polymerizing actin network could push p14 only in case it is impeded from sliding back by some counter-force. For example, in the case of the actin-driven movement of leading edge of a lamellipodium, the counter-force is supposed to be a friction force between the actin gel and the external substrate mediated by cell adhesions. The origin of the counter-force in the proposed mechanism should be discussed.

Moreover, the force could have a direction opposite to that schematized in Figure 5D if actin acts through V shape pulling such that the edges of the V stick out and bind to another membrane (for pushing versus pulling by polymerizing actin see, e.g. (Simon et al., 2019)).

Finally, the possible values of the pushing force have to be estimated and compared to what one needs to push p14 through the membrane gap.

# Reply: Author response

**DOI:** 10.7554/eLife.51358.sa2

Essential revisions:

The first group of questions, which have to be addressed, is related to the uniqueness of the proposed mechanism. Specifically, can N-WASP mediated assembly of a branched actin network promote fusion by raising local concentration of p14 at the future fusion site?

We agree that the question of whether fusion is driven by raising the local concentration of p14 at the fusion site is a very important one. As described below, we have carried out additional experiments to address this question based on the reviewers’ suggestions. More findings and details of the results are provided below.

Can one exclude fusion dependence on branched actin dependent cell-cell adhesion (Efimova and Svitkina, 2018)?

This is an interesting topic. A prior study found that p14-mediated cell-cell fusion depends on E-cadherin and branched actin assembly associated with E-cadherin (Salsman et al., 2008). However, the ability of p14’s cytoplasmic tail to nucleate branched actin network assembly was not known at the time. Our work demonstrates that branched actin network assembly, mediated by Grb2 binding to the cytoplasmic tail of p14, is essential for robust cell-cell fusion. In particular, we find that p14 FVAI, which cannot bind to Grb2 or nucleate N-WASP-mediated actin assembly, causes HEK293T cells to fuse less readily even though branched actin dependent cell-cell adhesion is intact. We further find that co-expression of p14 truncation mutants, Δecto and Δcyto, is also insufficient to drive cell-cell fusion, indicating that branched actin assembly at the cytoplasmic tail must be directly coupled to the fusogenic ectodomain of p14. This suggests that branched actin assembly at cell-cell adhesion is unable to functionally replace actin assembly that is directly coupled to p14, and we have added this point to the revised manuscript’s discussion. The question of how p14 and cell-cell adhesions might cooperatively drive cell-cell fusion is a very interesting one that we would like to pursue in the future, especially considering our observation that cell-cell fusion is not completely abolished by the p14 FVAI mutant. However, since a putative role for E-cadherin has already been published (Salsman et al., 2008) and a mechanistic analysis of p14-adhesion interactions would take considerable time, we feel this topic would be best addressed in a separate study.

Is there any evidence (perhaps in the literature) that fusion depends on interactions between p14 ectodomain and the target membrane rather than on the p14 ectodomain interactions with the p14-expressing membrane?

Yes, prior *in vitro* work demonstrated that soluble myristoylated p14 ectodomain is able to disrupt pure liposomes without being anchored in a bilayer as a membrane protein (Corcoran et al., 2004). Full-length p14 embedded in liposomes can also fuse with liposomes lacking p14 if close contact is first established using DOPS and divalent cations (Top et al., 2005). Together, this suggests that p14 ectodomain could interact with either the target membrane or the p14-expressing membrane, however close apposition is required to fuse the two membranes together. We have clarified this point in our revised manuscript.

Can one decrease the dependence of p14-mediated fusion on the assembly of the branched actin networks by "shaving" the surface of the target cells with proteases?

This is a very interesting suggestion that we also investigated. If cell surface proteins represent a steric barrier to cell-cell fusion, shaving the surface of the target cells with proteases would decrease the barrier and possibly decrease p14’s dependence on branched actin network assembly. To test this, we mildly trypsinized HEK293T cells and mixed them with p14-expressing HEK293T cells, and compared the extent of fusion with untreated cells. Unfortunately, the trypsinized cells did not adhere to each other and were unable to form cell-cell contacts needed for p14-mediated cell-cell fusion. This raises the important point discussed above about the role of E-cadherin and suggests a possible role is to adhere cells so that p14-driven cell-cell fusion can occur.

The reviewers realize that some of these questions are most likely beyond the scope of this study so that they can be addressed by mentioning alternative interpretations and avoiding the definitive language such as "we demonstrate" while discussing the proposed mechanism.

Thank you for the comment. We agree and have changed the language in our revised manuscript to be less definitive regarding the proposed mechanism.

At the same time, one of the alternative mechanisms has to be ruled out experimentally, namely, the mechanism in which the factor controlling fusion is the local concentration of p14, whereas the role of the polymerizing actin network is to modulate this concentration rather than to develop a pushing force. To address that point the reviewers suggest to:

1) Quantify the surface expression of p14 in wt and mutants under different experimental conditions and demonstrate that the differences in the fusion efficiency do not correlate with those in the p14 concentrations.

Addressing this point is especially important since differences in the number and density of fusogens on the surface can result in different levels of fusion. This has been shown for many fusogens including viral (e.g. Influenza's HA), exoplasmic (e.g. EFF-1) and endoplasmic (e.g. SNAREs). One of the reviewer suggested that the authors do surface biotinylation on ice for all the mutants under different pharmacological conditions (for example in Figures 1F, Figure 1—figure supplement 1B, Figure 2C, Figure 3B,C,E, Figure 4B, Figure 5A).

Thank you for the helpful suggestions of experiments. To test whether the extent of fusion we measured were correlated with differences in surface expression of p14 WT, p14 mutants and in p14 WT-expressing cells treated with wiskostatin and CK-666, we biotinylated cells expressing the p14 mutants with a cell-impermeable reagent (NHS-Biotin) on ice to limit endocytosis. We then immunoprecipitated biotinylated surface molecules with streptavidin beads and probed for p14 with α-GFP antibody (Author response image 1A). We calculated the ratio of intensity of bands eluted from the streptavidin beads to the intensity of the lysate. We then normalized this ratio for either cells expressing p14 WT or non-treated cells. While there is some variation, we found no statistically significant differences between surface expression of p14 FVAI, p14 Δcyto, p14 Δecto with that of p14 WT (Author response image 1B). We similarly found no statistically significant differences in surface expression of p14 in cells treated with wiskostatin or CK-666, and no trend that correlates with fusion efficiency (Author response image 1B). We conclude that the changes in fusion efficiency that we report are not due to changes in surface concentration of p14. We have added this in the revised manuscript as part of Figure 1—figure supplement 1H, Figure 3 —figure supplement 1A, Figure 4 —figure supplement 1A and B, Figure 5 —figure supplement 1A.

**Author response image 1.** Surface biotinylation of p14, p14 mutants and cells treated with cytoskeletal drugs. (**a**) Representative western blot probed with α-GFP of lysate and streptavidin eluate of surface biotinylated cells expressing p14 WT, p14 FVAI, p14 Δcyto, p14 Δecto, and p14 WT expressing cells treated with Wiskostatin and CK-666. (**b**) Ratio of streptavidin eluate to lysate of p14 FVAI, p14 Δcyto, p14 Δecto, and p14 WT expressing cells treated with Wiskostatin and CK-666 normalizd to that of p14 WT. Average and standard deviations from three independent transfections and blots shown. P-values are two-tailed, two-sample Student’s t-test between condition and p14 WT n.s p>0.05.

2) Perform knock down or knock out experiments using siRNAS or CRISPR-Cas9 in order to determine specifically whether Grb2, N-WASP, Arp2/3, Raf kinase and Ras are required for p14-mediated fusion.

We thank the reviewers for the suggested experiments. The results of our new experiments are presented below.

Quantification of p14-mediated cell-cell fusion in Grb2 KO and KD cells

In this work, we over-express the SH2 domain (58-159) of Grb2 to compete with endogenous Grb2 binding to p14. The SH2 domain alone does not bind to either downstream effectors, N-WASP or SOS, and is used in a dominant negative manner. Prior work have demonstrated the specificity of the SH2 domain of Grb2 for ligands (Jadwin et al., 2016; Kessels et al., 2002; Songyang et al., 1993). Moreover, in our system, the SH2 domain is quite specific to the p14 phosphorylation-dependent motif YVNI. To investigate the specificity of SH2 domain for p14 YVNI motif, we used a tyrosine phosphatase inhibitor, pervanadate, to broadly increase tyrosine phosphorylation (Figure 3—figure supplement 1G). In cells expressing p14 WT and treated with pervanadate, Grb2 enriches readily at the plasma membrane. However in cells treated with pervanadate, Grb2 does not enrich at the plasma membrane of wildtype HEK293T cells or in p14 FVAI-expressing cells (Figure 3—figure supplement 1G). Hence, over-expression of the SH2 domain likely specifically competes with Grb2.

As suggested by reviewers, we knocked-down Grb2 in HEK293T cells using shRNA (Author response image 2A). Surprisingly, these knocked-down cells expressing p14 still readily fused (Author response image 2B). We hypothesize that while Grb2 expression is knocked-down in the population overall, a fraction of individual cells still express Grb2 at sufficient levels to drive fusion. As p14-expressing cells fuse with neighboring cells, the cytoplasm mixes, re-introducing Grb2.

To overcome this, we knocked-out Grb2 using CRISPR-Cas9, and characterized 3 clones (Author response image 2C and D). Expression of p14 in two of these clones (clone 1 and clone 2) had minimal cell-cell fusion (Author response image 2E). Surprisingly, expression of p14 in the other Grb2 knock-out clone (clone 3) fused robustly, independent of Grb2 (Author response image 2D and E). Clone 3 might fuse robustly due to various reasons, including (i) increased adhesion between cells resulting in closer apposition of the bilayers than WT cells, (ii) decreased fusion energy barrier resulting in a lowered dependence on actin assembly, or (iii) upregulation of a compensatory pathway. We are interested in investigating the biophysical and molecular basis that enable this clone to fuse in a Grb2-independent manner. However, we think this would be best pursued in a future study. For this revised manuscript, we have made it clear that there are likely additional mechanisms contributing to p14-mediated cell-cell fusion.

**Author response image 2.** Extent of p14-mediated cell-cell fusion in Grb2 KD and KO cells. (**a**) Western blot probed with α-Grb2 of HEK293T with Grb2 knockdown. (**b**) Extent of p14-mediated cell-cell fusion in Grb2 KD and wild-type HEK293T cells. (**c**) Western blot probed with α-Grb2 of clone 1 and clone 2 of HEK293T with Grb2 knocked-out using Crispr-Cas9. (**d**) Western blot probed with α-Grb2 of clone 3 of HEK293T with Grb2 knocked-out using Crispr-Cas9. (**e**) Representative confocal images of wild-type HEK293T cells, KO clone 1, KO clone 2, KO clone 3 (magenta). Nuclei were stained with Hoechst 33342 (cyan).

Quantification of p14-mediated cell-cell fusion in N-WASP null cells.

To determine if N-WASP is specifically involved in p14-mediated cell-cell fusion, we expressed p14-WT in N-WASP null mouse embryonic fibroblasts (a gift of Scott Snapper). Since this is a different cell type than our other experiments, we compared the extent of cell-cell fusion to that of p14 expressed in wild-type mouse embryonic fibroblasts (MEFs). The overall transfection efficiency in MEFs was much lower than that of HEK293T, and the extent of cell-cell fusion was also lower. We quantified the extent of fusion as a percent of p14-expressing cells with 2 or more nuclei. When transfected with p14-WT-mCherry, cell-cell fusion was significantly decreased in N-WASP null MEFs than in the control MEFs. We have included this data as Figure 4D of the revised manuscript.

Quantification of p14-mediated cell-cell fusion in SYF (src, yes, fyn kinase) null cells

Since inhibition of Raf kinase with sorafenib had no effect on p14-mediated fusion, and our*in vitro* kinase results demonstrated that c-src kinase could phosphorylate Y116 in p14 cytoplasmic tail, we investigated the specificity of c-src kinase for Y116 in vivo. We compared the extent of cell-cell fusion in mouse embryonic fibroblasts lacking in c-src, Yes, Fyn kinase (SYF) and SYF + c-src cells expressing p14. Similar to the MEFs, the overall transfection efficiency in SYF cells were much lower than that of HEK293T, hence we quantified the extent of fusion as percent of p14-expressing nuclei with more than 2 nuclei. When transfected with p14-WT-mCherry, cell-cell fusion was more significantly decreased in SYF cells compared to SYF + c-src. We have included this data as Figure 2G of the revised manuscript.

3) Perform cell-cell imaging and colocalization of p14, actin and contact sites between membranes before during and after fusion can help to test the model.

We thank the reviewers for the suggested experiments. The results of our new experiments are presented below.

Live-cell imaging of p14 at fusion sites before, during and after fusion

We quantified the local concentration of p14 at sites of fusion using confocal microscopy in order to explore whether local concentration changes of p14 correlate with fusion. To visualize the plasma membrane we expressed a GPI-anchored pHluorin in p14-mcherry expressing cells and imaged every 20 seconds. Fusion sites were identified when a fusion pore expands and the plasma membrane peels back. We designated time of fusion as when the plasma membranes of two cells undergo full fusion and their cytoplasms mix. To identify the time when this diffraction-limited fusion pore opens, we used the cytoplasmic mCherry fluorophore from cleaved p14 as a marker of when the cytoplasm mixes between p14-mcherry expressing cell and a neighboring naive cell (Figure 5—figure supplement 5A). We then quantified the fluorescence intensity of p14-mcherry at the fusion site 200 seconds prior to cytoplasmic mixing and normalized it to the fluorescence intensity of p14-mcherry at another location on the plasma membrane. The normalized fluorescence intensity of p14-mcherry from 6 fusion events fluctuated around 1.0-1.1, indicating that p14-mcherry does not enrich at times and at sites of fusion, at least on the length scales resolvable by confocal microscopy (Figure 5—figure supplement 5B).

To test whether phosphorylated p14, in particular, might be locally enhanced during fusion, we expressed the GFP-tagged SH2 domain of Grb2 under a low-expressing promoter, the UBC promoter. We verified that this SH2 domain binds to phosphorylated p14 by treating p14-expressing cells with a broad tyrosine-phosphatase inhibitor, pervanadate. The GFP-tagged SH2 domain enriched at the plasma membrane, where p14 is localized to (Author response image 3). Using the cleaved mCherry fluorophore to again identify fusion pore opening, we quantified the fluorescence intensity of GFP-tagged SH2 domains at the fusion site 210 seconds prior to cytoplasmic mixing (Figure 5—figure supplement 5D). The normalized fluorescence intensity of SH2 domain from 5 fusion events also fluctuated around 1.0-1.1, indicating that phosphorylated p14 does not enrich at times and sites of fusion, at least on the length scales resolvable by confocal microscopy (Figure 5—figure supplement 5E). On average, we observed only a 10% increase in total and phosphorylated p14-mCherry, at times and sites of fusion. This is in contrast to previously reported plasma membrane enrichment, vesicle-docking and puncta-like structures of cell-cell fusogens, such as Eff-1 and Hap2, at fusion sites (Y. Liu et al., 2015; Neumann et al., 2015; Smurova and Podbilewicz, 2016; Y. Yang et al., 2017). This suggests that the p14-actin mechanism described in this manuscript does not require increased local concentration of p14 at fusion sites. We have included these results as Figure 5—figure supplement 2.

**Author response image 3.** Time-lapse imaging of p14 at sites of fusion using confocal microscopy. SH2-GFP (green) re-localizes to the plasma membrane when co-expressed with p14-WT mCherry (not shown) and treated with pervanadate to increase phosphorylation of p14.

Live-cell imaging of p14 and actin at fusion sites before, during and after fusion.

We carried out live imaging of cells expressing p14-WT-mCherry and actin with Lifeact-GFP to observe if there was co-localization of p14 with actin structures that would be expected from our proposed mechanism. We were able to capture 7 fusion events. To quantify p14 and actin colocalization, we measured the fluorescence intensity of Lifeact-GFP at the sites of fusion 210 seconds prior to cytoplasmic mixing and normalized. We were able to identify the site of fusion by determining where the fusion pore expands and the plasma membrane is pulled back.

For 6 of the fusion events, we did not observe actin structures at sites and times of fusion that can be readily identified as distinct from cortical actin structures of wild-type HEK293T cells (Author response image 4A and B). For 1 of the fusion events, we were able to capture increased local actin intensity at the site and time of fusion (Author response image 4B and C). However, we are unable to rule out that this was merely coincidental co-localization of fusion with endogenous actin assembly unrelated to fusion.

Given our*in vitro*evidence that the cytoplasmic tail of p14 can nucleate actin assembly, we were puzzled by the lack of co-localization of prominent actin structures and p14. However, we previously found that p14 Y116 was likely minimally phosphorylated (Figure 3—figure supplement F), suggesting that any nucleated actin structure was likely small, transient and difficult to distinguish from the endogenous cortical actin structures of the cells. Taken together, these experiments suggest that actin nucleation by p14, which must be phosphorylated at Y116, are likely sub-diffraction limited and transient, making them very difficult to identify separately from the endogenous actin structures.

**Author response image 4.** Time-lapse imaging of p14 and actin at times and sites of fusion. (**a**) Representative snapshots of a fusion site 90 sec prior to cytoplasmic mixing. Actin is visualized with Lifeact-GFP(green) and p14-WT mCherry in magenta. Boxed region marks the fusion site, where the average lifeact-GFP fluorescence intensity is quantified and normalized to the fluorescence intensity at a reference site on the plasma membrane. Representative fluorescent images of a fusion site 90 sec prior to cytoplasmic mixing. Actin is visualized with Lifeact-GFP(green) and p14-WT mCherry in magenta. Boxed region marks the fusion site, where the average lifeact-GFP fluorescence intensity is quantified and normalized to the fluorescence intensity at a reference site on the plasma membrane. (**b**) Normalized lifeact-GFP fluorescence intensity at the fusion site 210 sec prior to cytoplasmic mixing for 7 fusion events. Bolded line is average normalized intensity and filled in areas are the standard deviations. (**c**) Fluorescent images of a fusion site 90 sec prior to cytoplasmic mixing where Lifeact-GFP fluorescence intensity is enriched at the fusion site.

The second group of questions is concerned with the physical background of the mechanism by which polymerizing actin could exert a force sufficient to press p14 ectodomains against the target membrane.

As an experimental substantiation of the mechanism the reviewers expect a test where, for example, another nucleator is used, so that the actin network (and, hence, the force) that pushes the protein is changed. In addition, p14 could be made longer, with repetitive motifs, to see whether the distance between the protein ends matters.

As suggested by reviewers, we experimentally changed the actin nucleator associated with p14. Branched actin assembly can exert up to 5 nN or up to 1250 nN/μm^2^ (Bieling et al., 2016; Mueller et al., 2017; Parekh et al., 2005; Prass et al., 2006), while formin-nucleated filopodia can exert up to 10 pN (Cojoc et al., 2007). We chose to test whether a constitutively active formin, made by truncating the wGBD domain of mDia2, could be used in place of N-WASP to drive p14-mediated fusion. We over-expressed ΔGBD-mDia2 as a fusion protein with the Grb2 SH2 domain to bind to phosphorylated p14. When transiently expressed in cells with p14 WT, we observed filopodia-like structures consistent with previously reported mDia2 nucleation activity (Author response image 5) (C. Yang et al., 2007). To compete with endogenous Grb2 across the population of cells involved in fusion, we stably expressed and selected cells expressing this construct. Unfortunately, cells that survived selection expressed a truncated, functionally null protein that did not exhibit the filopodia-rich phenotype seen in the transient transfection. Due to the competition with endogenous Grb2 and minimal expression of the full-length SH2-nucleator, we were unable to conclusively show that swapping N-WASP for a different actin nucleator preserves p14-mediated fusion.

**Author response image 5.** Confocal image of HEK293T cell over-expressing SH2-GFP-ΔGBD-mDia2 and p14-WT-mCherry. HEK293T cell expressing SH2-GFP- ΔGBD-mDia2 (green) and p14-WT-mCherry (magenta) with filopodia-like protrusions denoted with white arrows.

On the theoretical level, the way the force pushing p14 is generated is not clear. Indeed polymerizing actin network could push p14 only in case it is impeded from sliding back by some counter-force. For example, in the case of the actin-driven movement of leading edge of a lamellipodium, the counter-force is supposed to be a friction force between the actin gel and the external substrate mediated by cell adhesions. The origin of the counter-force in the proposed mechanism should be discussed.

Moreover, the force could have a direction opposite to that schematized in Figure 5D if actin acts through V shape pulling such that the edges of the V stick out and bind to another membrane (for pushing versus pulling by polymerizing actin see, e.g. (Simon et al., 2019)).

This is a good point, as simply the assembly of a branched network doesn’t guarantee formation of a protrusion. From the in vitro assay of Simon et al., 2019, based on actin polymerization from the surface of a GUV, the ability to deform the membrane is dependent on membrane tension. We have also seen similar spike-like membrane protrusions driven by branched actin networks assembled on the surface of GUVs in our own in vitro work (A. P. Liu et al., 2008). This model of membrane protrusions driven by local actin assembly that is coupled to cortical actin has been proposed for other WASP-dependent membrane protrusions in fusogenic synapses (Sens et al., 2010) and T-cell protrusions (Tamzalit et al., 2019). We hypothesize that p14-mediated actin polymerization will be coupled to and countered by the stiff cortical actin underneath the network, providing a counter-force that allows it to generate local membrane protrusions. However, it is possible that p14 will, on occasion, not form branched actin networks that are strongly coupled to the cortex, and we expect that these p14 will not be successful in driving cell-cell fusion. To clarify this, we have updated the revised manuscript to emphasize that the direction of force in our model is a hypothesis.

Finally, the possible values of the pushing force have to be estimated and compared to what one needs to push p14 through the membrane gap.

This is a good suggestion to see if the forces generated by p14 could be enough to overcome the membrane gap. First, to estimate the maximum force that p14 can generate, we use the measured stall force of branched actin assembly. We previously measured the force generated by Arp2/3 branched actin networks nucleated by WASP-family NPF in vitro and found the stall force to be approximately 1250 pN/µm^2^ (Bieling et al., 2016; Parekh et al., 2005). This is consistent with the stall force measured for a migrating keratocye (Mueller et al., 2017; Prass et al., 2006).

Next, we estimated the force that would be required to expose a patch of bare lipid bilayer at the site of fusion. Membrane proteins on a cell surface exert in-plane pressure due to protein-protein crowding, which we previously found to be sufficient to drive membrane tubulation (Stachowiak et al., 2012). In order for membranes to make close contact for fusion, these membrane proteins must be excluded. Assuming that proteins on a cell surface cover between 0.2 to 0.3 of the surface area and the projected area of average protein is 100 nm^2^, the in-plane pressure exerted by crowded proteins is between 3100 kT/µm^2^ to 6300 kT/µm^2^, based on a 2D Carnahan-Starling equation of state for hard discs (Stachowiak et al., 2012).

For an actin protrusion to successfully bring one membrane in contact with the other, the force generated by an actin-based protrusion must do enough work to overcome the in-plane pressure calculated above. Assuming an initial intermembrane distance of 20 nm (accounting for two cell surfaces with average cell surface protein heights of ~10 nm) and that the actin protrusion is pushing directly on the patch of membrane that must be cleared for fusion to take place at 500-1250 pN/ µm^2^, we estimate the work done by actin is between 2400 kT/µm^2^ to 6000 kT/µm^2^. This simple calculation suggests that branched actin network can provide a force that is sufficient to segregate proteins out of the membrane contact site.
